# Clinical heterogeneity of frontotemporal dementia and Parkinsonism linked to chromosome 17 caused by *MAPT* N279K mutation in relation to tau positron emission tomography features

**DOI:** 10.1002/mds.27623

**Published:** 2019-02-17

**Authors:** Aya Ikeda, Hitoshi Shimada, Kenya Nishioka, Masashi Takanashi, Arisa Hayashida, Yuanzhe Li, Hiroyo Yoshino, Manabu Funayama, Yuji Ueno, Taku Hatano, Naruhiko Sahara, Tetsuya Suhara, Makoto Higuchi, Nobutaka Hattori

**Affiliations:** ^1^ Department of Neurology Juntendo University School of Medicine Tokyo Japan; ^2^ Department of Functional Brain Imaging Research National Institute of Radiological Sciences, National Institutes for Quantum and Radiological Science and Technology Chiba Japan; ^3^ Research Institute for Diseases of Old Age, Graduate School of Medicine Juntendo University Tokyo Japan

**Keywords:** frontotemporal dementia, MAPT, N279K mutation, tau PET

## Abstract

**Background:**

While mechanistic links between tau abnormalities and neurodegeneration have been proven in frontotemporal dementia and parkinsonism linked to chromosome 17 caused by MAPT mutations, variability of the tau pathogenesis and its relation to clinical progressions in the same MAPT mutation carriers are yet to be clarified.

**Objectives:**

The present study aimed to analyze clinical profiles, tau accumulations, and their correlations in 3 kindreds with frontotemporal dementia and parkinsonism linked to chromosome 17 attributed to the *MAPT* N279K mutation.

**Methods:**

Four patients with N279K mutant frontotemporal dementia and parkinsonism linked to chromosome 17/*MAPT* underwent [^11^C]PBB3‐PET to estimate regional tau loads.

**Results:**

Haplotype assays revealed that these kindreds originated from a single founder. Despite homogeneity of the disease‐causing *MAPT* allele, clinical progression was more rapid in 2 kindreds than in the other. The kindred with slow progression showed mild tau depositions, mostly confined to the midbrain and medial temporal areas. In contrast, kindreds with rapid progression showed profoundly increased [^11^C]PBB3 binding in widespread regions from an early disease stage.

**Conclusions:**

[^11^C]PBB3‐PET can capture four‐repeat tau pathologies characteristic of N279K mutant frontotemporal dementia and parkinsonism linked to chromosome 17/*MAPT*. Our findings indicate that, in addition to the mutated *MAPT* allele, genetic and/or epigenetic modifiers of tau pathologies lead to heterogeneous clinicopathological features. © 2019 The Authors. Movement Disorders published by Wiley Periodicals, Inc. on behalf of International Parkinson and Movement Disorder Society.

Tau protein fibrillation has been implicated in Alzheimer's disease (AD), frontotemporal lobar degeneration (FTLD) subtypes and related disorders, which are collectively referred to as tauopathies.^1^ FTLD tauopathies, including PSP and corticobasal degeneration (CBD), are characterized by the deposition of four‐repeat tau isoforms in neurons, astrocytes, and oligodendrocytes.^2^ Distinct tau isoforms cause ultrastructural and conformational diversity of the pathological fibrils, represented by paired helical filaments in AD and straight filaments in PSP and CBD.^3^

Despite the association between tau conformers, localization of tau lesions, and clinical phenotypes, the symptomatic manifestations and progression of a single tauopathy can vary.^4^, ^5^, ^6^ The microtubule‐associated protein tau (*MAPT*) haplotypes may account for the clinicopathological characteristics of PSP^7^ and frontotemporal dementia (FTD).^4^, ^8^ Moreover, a number of *MAPT* mutations cause familial tauopathies, which are termed frontotemporal dementia and parkinsonism linked to chromosome 17 *MAPT* (FTDP‐17/*MAPT*). However, the symptomatic profiles of patients carrying identical *MAPT* mutations are also variable.^9^, ^10^, ^11^, ^12^

Evaluation of the correlation between the clinical course and chronological sequence of regional pathological involvement has been enabled by in vivo PET of tau lesions in humans. The radioligand, [^11^C]pyridinyl‐butadienyl‐benzothiazole 3 ([^11^C]PBB3), binds to a wide range of tau fibrils, including AD, PSP, and putative CBD tau deposits.^13^, ^14^, ^15^ Other tracers, such as [^18^F]AV‐1451, produce a higher contrast for AD‐type tau tangles than it does for four‐repeat tau inclusions in PSP and CBD,^16^, ^17^ although [^18^F]AV‐1451 has enabled differentiation between groups of PSP patients and healthy controls.^18^ The distinct selectivity of the PET ligands could help identify tau isoforms contributing to unique neurodegenerative pathologies in each individual.^19^

The *MAPT* N279K mutation was discovered in the white pallidopontonigral degeneration (PPND) kindred^20^ and was also found in 6 Japanese kindreds.^21^, ^22^, ^23^ In the present work, we further identified two novel Japanese families with hereditary tauopathy caused by the N279K mutation, and we investigated the abundance and extent of tau deposits in patients harboring the *MAPT* N279K mutation derived from three pedigrees, including these two families. Because our previous in vitro assays demonstrated binding of [^11^C]PBB3 to N279K mutant four‐repeat tau aggregates,^19^ [^11^C]PBB3‐PET allowed us to analyze fibrillary tau pathologies in living patients in these families. The haplotypes of all mutant *MAPT* allele carriers examined here were identical, presumably originating from a single founder. However, there was a profound difference in the progression of functional impairments among these 3 kindreds, in close association with the severity of PET‐detectable tau pathologies.

## Patients and Methods

### Participants

The current study was approved by the local ethics committees of the Juntendo University School of Medicine and National Institute of Radiological Sciences, of which the registration numbers of UMIN are #000009863 and #000017978. All participants or caregivers were fully informed and provided written consent. Verbal ascent was obtained from demented patients and was confirmed by their caregivers. We enrolled 10 patients with FTDP‐17 attributed to *MAPT* N279K mutation, and 6 of these patients were derived from 2 newly identified kindreds (families A and B; Supporting Information Table S1, Supporting Information Fig. S1, and Supporting Information Case Presentation). Procedures to analyze their *MAPT* genes are provided in the Supporting Information Materials and Methods. Four participants were derived from a pedigree reported on previously (designated family C in the resent study and “family D” in our earlier report^23^; Supporting Information Table S1, Supporting Information Fig. S1, and Supporting Information Case Presentation). We also included 13 age‐ and sex‐matched cognitively healthy controls (HCs), who have been already confirmed as having a negative [^11^C]Pittsburgh Compound‐B ([^11^C]PiB) PET scan in our previous study.^10^

### Tau and Amyloid PET Imaging

PET scans with [^11^C]PBB3 and [^11^C]PiB were performed on 4 patients (A‐II‐1, B‐II‐2, C‐IV‐1, and C‐IV‐2) to estimate regional tau and amyloid‐β loads, respectively, as described in the Supporting Information Materials and Methods. Two patients received scans within 1 year of clinical onset of the disease, whereas the other 2 patients underwent scans relatively late. We generated parametric images of the standardized uptake value ratio (SUVR) for [^11^C]PBB3 and [^11^C]PiB at 30 to 50 and 50 to 70 minutes, respectively, after radioligand injection, using the cerebellar cortex as a reference region. To estimate local tau and amyloid‐β burdens, we performed volume of interest (VOI)‐based quantifications of SUVRs for a group analysis, and conducted a voxel‐by‐voxel jack‐knife examination of parametric SUVR images to statistically assess distributions of areas with an increased [^11^C]PBB3 retention in each patient compared to 13 HCs. Detailed analytical procedures are provided in the Supporting Information Materials and Methods.

## Results

### Clinical and Genetic Profiles

Clinical and genetic characteristics of all 10 patients are summarized in Supporting Information Table S1 and Supporting Information Figure S1, and detailed clinical information of all patients and family members is described in the Supporting Information Case Presentation. Despite the haplotypic homogeneity of the mutant *MAPT* allele among the patients, Kaplan‐Meier analysis depicted significant differences in the survival proportions between combined A and B families and family C (*P* = 0.01 by log‐rank test; Supporting Information Fig. S1C). Members of family C had a better prognosis than those of families A and B.

### PET Imaging

**Figure 1:**
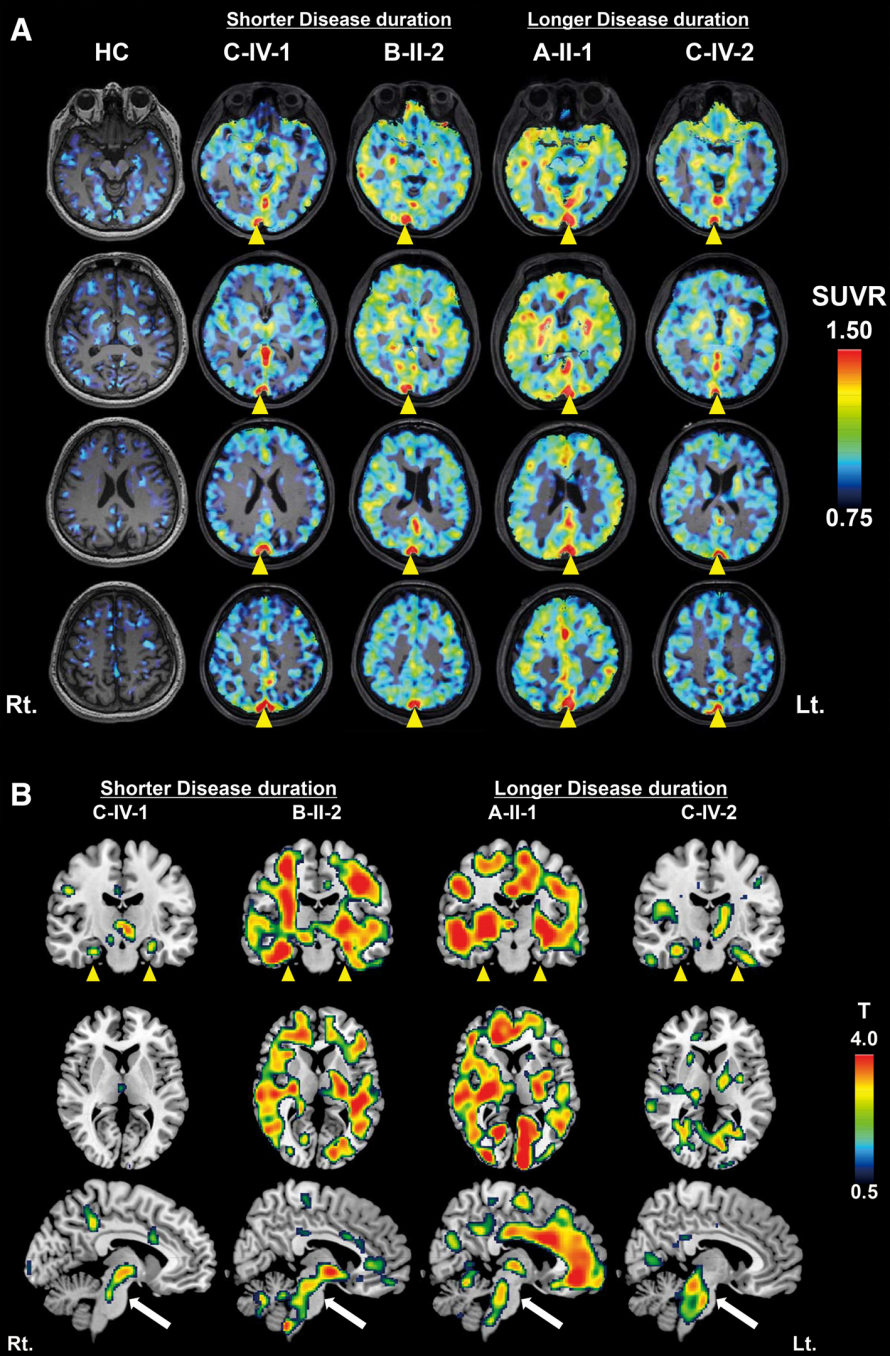
[^11^C]PBB3‐PET images of representative cognitively HCs and patients with N279K mutant FTDP‐17/*MAPT* and voxel‐based comparison of [^11^C]PBB3 SUVR between each patient and control group. (A) Axial parametric SUVR images, acquired at 30 to 50 minutes after radioligand injection, were superimposed on the corresponding MR images. All patients showed noticeable uptake of [^11^C]PBB3 in multiple brain regions and the superior sagittal sinus (yellow arrowheads). (B) Localization of increased [^11^C]PBB3 SUVR in each patient compared with HCs was highlighted in coronal (top), axial (middle), and sagittal (bottom) SPM t‐maps. A patient with the shortest disease duration (C‐IV‐1) already showed remarkable enhancement of [^11^C]PBB3 binding in several areas, including the midbrain (white arrows) and medial temporal cortex (yellow arrowheads). Members of families A and B exhibited more extensive [^11^C]PBB3 radiosignals, particularly in neocortical gray and white matter, than cases derived from family C. [Color figure can be viewed at wileyonlinelibrary.com]

Compared to HCs, all scanned patients had larger [^11^C]PBB3 SUVRs in characteristic brain regions, including neocortical gray and white matter (Table 1; Fig. 1A). This was distinct from the gray matter–dominant topology of tau depositions in the AD spectrum^13^, ^14^ and corresponded to previous [^11^C]PBB3 autoradiographical findings.^19^ Subject C‐IV‐1 had the shortest interval between onset and PET scans and exhibited a remarkable increase of [^11^C]PBB3 SUVRs in the midbrain, including the SN, hippocampus, and amygdala, suggesting that tau pathologies could arise from these regions (Fig. 1A). Tau deposits appeared to expand from the brainstem and limbic areas to the neocortex and subcortical nuclei with disease progression, given that subject C‐IV‐2, who underwent PET assays 4 years after onset, presented more widespread and greater increase of [^11^C]PBB3 bindinginvolving neocortical white matter, globus pallidus, and thalamus than subject C‐IV‐1 (Table 1).

**Table 1:** [^11^C]PBB3‐PET data in subjects A‐II‐1, B‐II‐2, C‐IV‐1, and C‐IV‐2 in comparison to HCs [Color table can be viewed at wileyonlinelibrary.com]

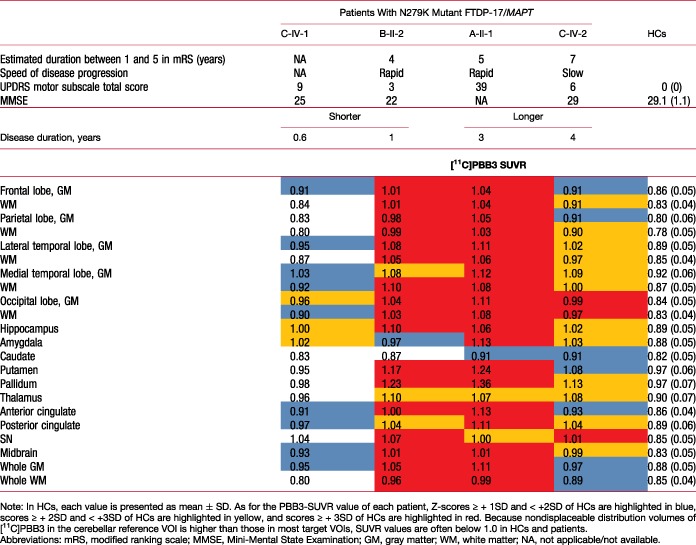

In line with the notable difference in the rate of progression to death between families A/B and C, a subject from family B (B‐II‐2), who was scanned 12 months after onset, had even higher levels of [^11^C]PBB3 retentions in most VOIs than subject C‐IV‐2, despite the relatively early stage of the clinical course (Fig. 1A). Radioligand binding in subject A‐II‐1, a member of family A undergoing PET examinations 3 years after onset, was comparable with that of subject B‐II‐2 in the majority of VOIs, although additional increases of [^11^C]PBB3 SUVRs were noted in several areas, including the parahippocampal gyrus and amygdala (Table 1). Therefore, PET‐visible tau pathologies in families A and B seemingly plateaued early during clinical progression. None of the patients were amyloid‐β positive according to visual and quantitative assessments of [^11^C]PiB‐PET data, which were conducted as in previous studies.^10^

In order to highlight areas with increased [^11^C]PBB3 retentions on brain maps, we also conducted voxel‐based statistical assessments of SUVR images for this tracer. SPM t‐maps depicted enhanced [^11^C]PBB3 radiosignals rather confined to the brainstem and a few other regions, including the hippocampus in family C, which was in sharp contrast with increases of radioligand binding in extensive areas containing neocortical gray and white matter in families A and B (Fig. 1B). This familial difference was observed in subjects with both short and long durations, notwithstanding that areas highlighted in the SPM maps were somewhat increased in a manner dependent on the disease duration.

## Discussion

We documented three Japanese families with the N279K FTDP‐17/*MAPT* mutation originating from a single founder according to a haplotype analysis. Two of these kindreds (A and B) are newly identified and are characterized by markedly rapid clinical progression, leading to death within 5 years of disease onset. In contrast, the third kindred (family C) showed relatively slow clinical progression with an approximate postonset survival period of 10 years. Hence, the present data illustrated a pronounced interfamilial difference in the aggressiveness of the illness, despite the similarity of their mutant *MAPT* allele.

Previous studies reported that patients with FTDP‐17/*MAPT*, which could be linked to the same single mutation, demonstrated inter‐ and intrafamilial heterogeneity in clinicopathological features, including ages at onset and death, disease duration, clinical symptoms, brain atrophy, and pathological findings.^9^, ^10^, ^12^, ^24^ Taken together with the present results, these observations support the view that the *MAPT* mutation alone may not fully define the clinical and neuropathological outcomes, which could in fact be modulated by other genetic and/or environmental components.

The PET results of the present study provide the first demonstration of heterogeneous neuroimaging phenotypes among patients with FTDP‐17/*MAPT* who possess the same pathogenic mutation and *MAPT* allele haplotype. In close association with clinical progress, affected cases in families A and B exhibited extensive increases of [^11^C]PBB3 binding in neocortical and subcortical areas from an early period after onset. Enhancement of [^11^C]PBB3 binding, however, was less prominent in patients from family C, who had a longer clinical duration than those from the other two families. These findings indicated that the formation of tau lesions in families A and B occurred rapidly at the perionset stage and then almost plateaued at an early postonset stage. This was then followed by a prompt evolution of functional deteriorations, resulting in a short life span of the affected members after onset. This may also suggest the significance of tau PET as a predictor of the following neurodegenerative processes, resembling findings in patients with AD, who show a tight correlation between baseline retention of a tau PET probe and subsequent longitudinal atrophy of the cortex.^25^

The symptomatic profiles of the current N279K mutant cohort were all PSP like, consistent with previous studies.^26^ However, two patients from family A initially presented personality changes (Supporting Information Table S1), raising the possibility that there is a variable chronology of neuropsychiatric phenotypes within pedigrees of a common origin. Similar diversities were also noted in members of PPND and Italian families with the N279K mutation^27^ and were conceived to stem from the H1/H2 haplotypes of *MAPT*.^28^ Given that the Japanese population does not possess the H2 haplotype,^29^, ^30^ the personality‐related presentation of initial symptoms observed in family A, but not in the other two families, could be attributed to additional genotypic variations located on the nonmutant *MAPT* allele and/or non‐*MAPT* elements.

Parkinsonian symptoms in affected individuals from family C from an early clinical stage are typical of the N279K mutation^26^ and could be induced by involvement of the extrapyramidal tract in tau pathologies. Indeed, a profound increase of [^11^C]PBB3 binding in subject C‐IV‐1 with a short postonset duration was particularly evident in the SN (Table 1), which might be an initiation site of tau fibrillogenesis at a preclinical stage. This may be in line with our previous PET findings, where the nigrostriatal dopaminergic system was disrupted in presymptomatic carriers of the N279K mutation derived from the PPND pedigree.^29^ Meanwhile, the origin of tau depositions in members of family A with initial manifestations dominated by psychiatric signs has yet to be clarified. The tau PET data of subject C‐IV‐1 (in the current study) also suggest that tau pathologies in the amygdala and hippocampal formation emerge early during the clinical course. This might elicit local neuronal death and atrophic changes, as illustrated by an MRI analysis of the above‐mentioned N279K mutant carriers at a prodromal disease stage.^29^

Similar to the AD spectrum,^30^ the extent of tau pathologies may reflect the disease progression in N279K mutant cases. However, the tau pathogenesis, even in family C, appeared to be rapidly progressive relative to AD. Moreover, regions and voxels with increased [^11^C]PBB3 binding in neocortical white matter of mutation carriers from all three families expanded over time, which differed from the gray matter–predominant distribution of tau fibrils in AD. Deposition of tau assemblies in white matter may be a neuropathological characteristic of familial^31^, ^32^ and sporadic^33^, ^34^ FTLDs with an excess of insoluble four‐repeat tau isoforms.

A few technical issues need to be considered in the interpretation of the current PET data. *In vivo* off‐target binding and nonspecific retention of [^11^C]PBB3 remain undetermined. Our recent in vitro binding assays using human brain homogenates have indicated that [^11^C]PBB3 does not cross‐react with monoamine oxidases A and B,^35^ which is in clear distinction from properties of other tau radioligands, including [^18^F]AV‐1451^36^ and [^18^F]THK5351.^37^ This observation, however, does not fully ensure the selectivity of [^11^C]PBB3 for tau fibrils in PET imaging of living patients with tauopathies. Another methodological issue is that tau deposits might exist in a portion of the reference VOI defined in cerebellar gray matter. This might occur in a case with severe and widespread tau accumulations as exemplified by subject A‐II‐1 (indicated in a sagittal map of Fig. 1B), potentially leading to underestimation of radioligand SUVRs in target areas and voxels.

In conclusion, the current study delineated the neuropathological basis of the clinical phenotypes in living patients with FTDP‐17/*MAPT*, underscoring the contribution of factors beyond the disease‐causative *MAPT* haplotypes and mutations to prompt the spread of tau and clinical progress. Although these modifiers are still unidentified, there could be common accelerators or decelerators of tau pathologies across a wide range of tauopathies. Moreover, our imaging assay has supported the significance of the baseline extent of tau lesions at an early clinical stage as a predictor of rapid and slow subsequent disease progressions. In the event that future clinical assays demonstrate that this can be translated to other four‐repeat tauopathies, tau PET would help to stratify an observational or interventional cohort of participants, based on an expected rate of clinical and pathological advancements.

## Author Roles

(1) Research Project: A. Conception and Design, B. Acquisition of Data, C. Analysis and Interpretation of Data; (2) Manuscript: A. Writing of the First Draft, B. Review and Critique; (3) Other: A. Drafting of Figures.

A.I.: 1B, 1C, 2A, 3A

H.S.: 1A, 1B, 1C, 2A, 3A

K.N.: 1A, 1B, 1C, 2A, 3A

M.T.: 1B, 1C, 2A, 3A

A.H.: 1B

Y.L.: 1B

H.Y.: 1B

M.F.: 1B

Y.U.: 1B

T.H.: 1B

N.S.: 1B

M.H.: 1B, 1C, 2A

T.S.: 2A, 2B

N.H.: 2A, 2B

## Financial Disclosures

Nothing to report.

## Supporting information


**Supplementary Figure 1** Genetic and clinical profiles of FTDP‐17‐*MAPT* patients derived from three families with the N279K *MAPT* mutation

**Appendix S1** Supporting information
