# Supplementary figures and images for: Clinical heterogeneity of frontotemporal dementia and Parkinsonism linked to chromosome 17 caused by MAPT N279K mutation in relation to tau positron emission tomography features
Source: Mov Disord. 2019 Feb 17;34(4):568–74. doi: 10.1002/mds.27623 (PMC6593784; doi:10.1002/mds.27623)

Figure 1

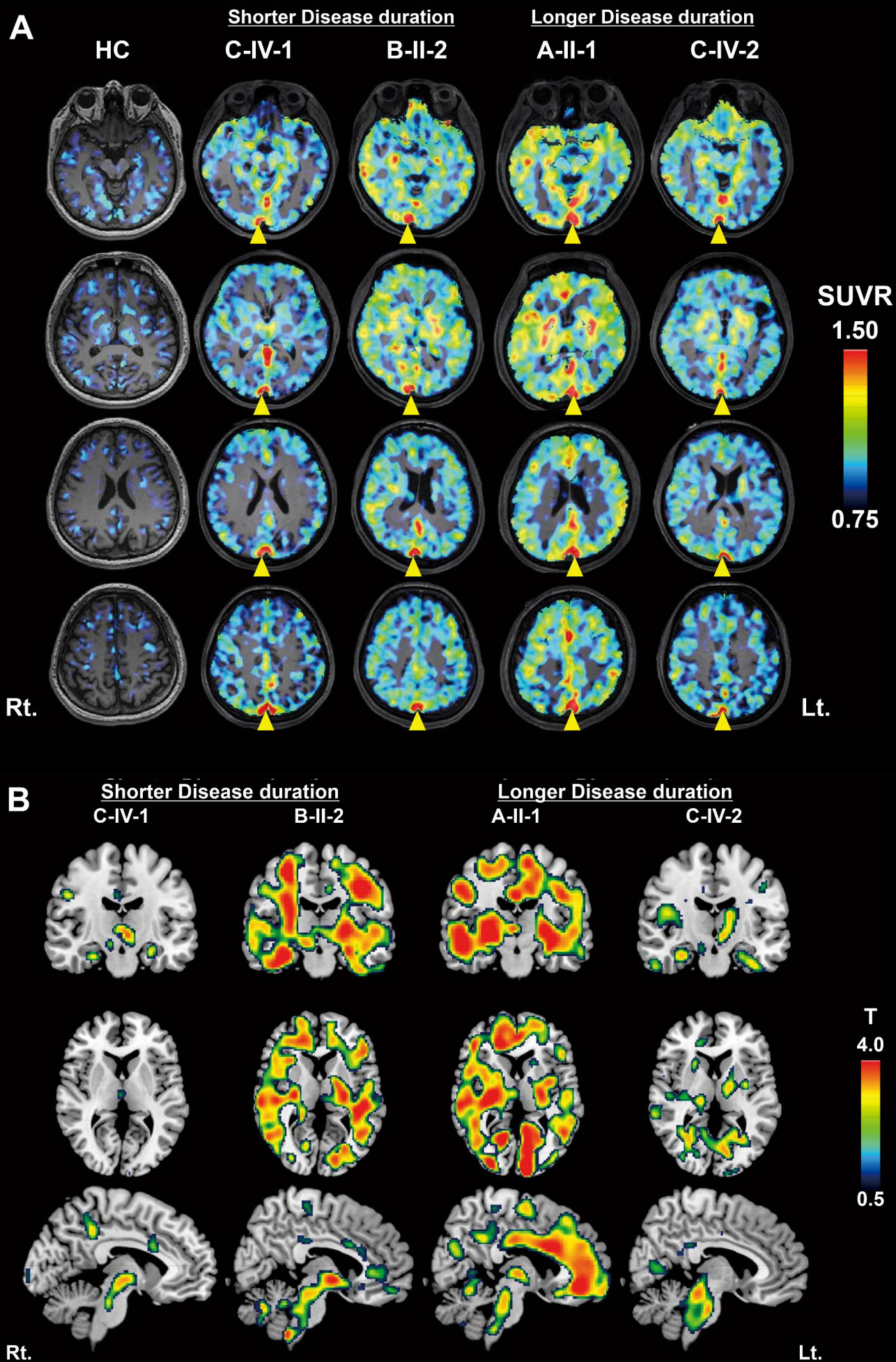

Supplement: Supplementary file 1 — Supplementary Figure 1 Genetic and clinical profiles of FTDP‐17‐MAPT patients derived from three families with the N279K MAPT mutation [file MDS-34-568-s001.pdf]
