# Supplementary material for: Clinical heterogeneity of frontotemporal dementia and Parkinsonism linked to chromosome 17 caused by MAPT N279K mutation in relation to tau positron emission tomography features
Source: Mov Disord. 2019 Feb 17;34(4):568–74. doi: 10.1002/mds.27623 (PMC6593784; doi:10.1002/mds.27623)
Supplement: Supplementary file 2 — Appendix S1 Supporting information [file MDS-34-568-s002.docx]

**Supplementary data**

**Supplementary Case Presentation**

**Family A**

A-II-1: At the age of 42 years, the patient was frequently falling down, and he noticed a tremor in his right lower limb. He was diagnosed with Parkinson’s disease (PD) in another hospital, presenting with rigidity, akinesia, resting tremor, and decreasing of facial expression. His temperament changed, with violent behavior becoming more prominent toward his family. At the age of 44 years, he was admitted to Juntendo University Hospital. He manifested rigid-akinesia Parkinsonism and prominent psychosis of visual hallucination, delusion, and irritability. Cognitive test indices were 29/30 in the Mini-Mental State Examination (MMSE), and 17/18 in the frontal assessment battery (FAB). Brain MRI indicated severe atrophic changes in the temporal lobe and parahippocampal gyrus. ^1-3^ I-FP-CIT dopamine transporter (DAT) scan, which was conducted using single photon emission computed tomography (SPECT), indicated severe reduction of specific binding ratio (SBR) as follows: right = 0.92, left = 0.87. Three-dimensional stereotactic surface projection (3D-SSP) analysis of brain SPECT showed hypoperfusion in the frontotemporal lobe.

A-I-4: Parkinsonism symptoms appeared in the patient at the age of 54 years, and cognitive decline became exacerbated the following year. At the age of 56 years, the patient had a fall and died of head trauma.

A-I-10: The patient experienced Parkinsonism at the age of 40 years. He died 1 year later.

A-II-3: The patient began to manifest Parkinsonism symptoms at the age of 38 years. He harbored *MAPT* N279K, which was proven by our genetic test. Body weight loss soon became prominent (-20 kg / 2 years). The patient died in the bath 3 years after disease onset.

**Family B**

B-II-2: The patient noticed akinesia in the right upper and lower limbs at the age of 40 years. He had difficulty swallowing 6 months after disease onset, which is when he attended Juntendo University Hospital. He presented with rigidity on the right side against levodopa treatment and progressive cognitive decline at the initial examination. The indices of cognitive tests were as follows: 16/18 by FAB and 23/30 by MMSE. He also had prominently complicated motor aphasia, which was categorized as primary progressive aphasia. His mood was always euphoric and calm, without psychosis or violent tendencies. Two years after the first examination, the scores were exacerbated to 9/18 and 20/30 by FAB and MMSE, respectively. His Parkinsonism had also rapidly worsened during the first 2 years. Brain MRI indicated severe atrophic changes in the temporal lobe and parahippocampal gyrus. A DAT scan indicated severe reduction of SBR; right=0, left=0. 3D-SSP analysis of brain SPECT demonstrated hypoperfusion in the frontotemporal lobe, with prominence on the left side. After admission to our hospital, he lived in the faculty. At the age of 44, he was found with cardiopulmonary arrest; the cause of death was unknown.

B-I-2: The patient manifested Parkinsonism from the early fifth decade. She died at age 55. Her cause of death was unknown due to a lack of medical information.

**Family C**

C-IV-1: The patient noticed akinesia in the right lower limb at the age of 34 years. The year after akinesia, tremor emerged in the right upper limb. Her two aunts (C-III-3 and C-III-4) were pathologically confirmed as having frontotemporal dementia, with proven N279K mutation. Thus, we assessed and confirmed that C-IV-1 was also positive for *MAPT* N279K. Brain MRI indicated mild atrophic changes in the temporal lobe. A DAT scan indicated severe reduction of SBR; right=1.32, left=0. 3D-SSP analysis of brain SPECT showed hypoperfusion in the bilateral frontal lobe. At age 39, her cognitive test indices indicated 12/18 by FAB. At 40 years, her cognitive decline had exacerbated. She always needed help when she walked and she had marked aphasia. She often just whispered and found it difficult to communicate with others. Her modified rating scale changed to 5.

C-IV-2: The patient presented with akinesia in the right upper and lower limbs at Juntendo University Hospital at the age of 44 years. At the first neurological examination, she could communicate with others; she showed no signs of cognitive decline or verbal problems. She showed rigidity and akinesia in her right upper and lower limbs. Her Hoehn and Yahr stage was I. The test indices related to cognitive function were 30/30 by MMSE and 15/18 by FAB. Brain MRI indicated no atrophic changes. DAT scan indicated severe reduction of SBR on the left side; right=2.57, left=0.55. Generally, she showed mild Parkinsonism. She underwent [^11^C]PBB3 PET analysis 5 months after disease onset.

C-I-1, C-II-3, C-II-5, C-II-6, and C-III-2 were diagnosed with Parkinson’s disease or atypical Parkinsonism during their lifetime. Details of the respective cases are unknown.

**Supplementary Materials and Methods**

**DNA analysis**

Genomic DNA was extracted from peripheral blood using standard protocols. DNA was amplified using direct PCR and then sequenced using the Sanger method, with a BigDye Terminators v1.1 Cycle Sequencing Kit and 3130 Genetic Analyzer (Life Technologies, Foster City, CA, USA). All coding exons and exon-intron boundaries of exons 1 to 10 of *MAPT* were screened. Sequences and PCR conditions have been described in detail in our previous reports.^26^

**Haplotype analysis**

Haplotype analyses of *MAPT* in seven probands (two cases from family A, one case from family B, and four cases from family C) were performed using seven microsatellite makers (D17S805, D17S798, D17S800, D17S810, D17S806, D17S797 and D17S809), five single nucleotide polymorphisms (SNPs) (rs1467967, rs242557, rs3785883, rs2471738 and rs7521), and an intronic microdeletion (del-in9). Alleles were sized using the GeneMapper (Life Technologies, Carlsbad, CA, USA). A total of 5 SNPs and del-in9 were analyzed using direct Sanger sequencing.

**Radiosynthesis and PET scan**

Radiosynthesis of [^11^C]PBB3 and [^11^C]PiB was conducted as described elsewhere.^a, b^ Patients underwent dynamic three-dimensional PET scans, at 50 and 70 min after intravenous injections of [^11^C]PBB3 (injected dose, 454 ± 79 MBq; molar activity at injection, 104 ± 77 GBq/μmol; chemical purity, 97.1 ± 0.6%) and [^11^C]PiB (injected dose, 415 ± 75 MBq; molar activity, 70 ± 7 GBq/μmol; chemical purity, 98.8 ± 0.7%), to evaluate tau and Aβ accumulations, respectively. PET data were acquired using a Siemens ECAT EXACT HR+ scanner (CTI PET Systems, Inc., Knoxville, TN), with an axial field of view of 155 mm, providing 63 contiguous 2.46-mm slices with 5.6-mm transaxial and 5.4-mm axial resolutions. Images were then reconstructed using the filtered back-projection algorithm (Hanning filter; cut-off frequency, 0.4 cycle/pixel) to secure methodological consistency with our previous clinical PET works with [^11^C]PBB3.^13, 14^ Attenuation and scatter corrections were applied to these images using the data of a 10-min transmission scan, with a 68Ge-68Ga line source and single-scatter simulation method, respectively. Three-dimensional T1-weighted magnetic resonance images (repetition time range/echo time range, 7 ms/2.8 ms; field of view [frequency × phase], 260 × 244 mm; matrix dimension, 256 × 256; 170 contiguous axial slices of 1.0 mm thickness) were acquired with a 3-T MRI scanner (Signa HDx; GE Healthcare, WI, USA, or MAGNETOM Verio, Siemens Healthcare, Erlangen, Germany) on the same day as the [^11^C]PBB3-PET scan.

All images were preprocessed using PMOD software version 3.8 (PMOD Technologies Ltd., Zürich, Switzerland) and Statistical Parametric Mapping software (SPM12, Wellcome Department of Cognitive Neurology, London, UK), operating in the MATLAB software environment (version 9.2; MathWorks, Natick, MA, USA). Data preprocessing and data analysis of the PET images were performed as previously described.^14^ Briefly, each PET image was co-registered to individual T1-weighted magnetic resonance images after motion correction, and anatomically normalized into Montreal Neurological Institute standard space (MNI152; Montreal Neurological Institute, Montreal, QC, Canada) using Diffeomorphic Anatomical Registration Through Exponentiated Lie Algebra (DARTEL).^c^ We placed a reference volume of interest (VOI) containing cerebellar gray matter, and this VOI did not include deep cerebellar structure, in consideration of tau depositions in the cerebellar dentate nucleus of some patients with 4-repeat tauopathies. We then generated parametric images of the standardized uptake value ratio (SUVR) for [^11^C]PBB3 and [^11^C]PiB at 30–50 and 50–70 min, respectively, after radioligand injection, using the cerebellar reference VOI. To estimate local tau and Aβ burdens, template VOIs were defined in several neocortical and subcortical regions, including gray and white matter of the frontal, parietal, occipital, medial and lateral temporal lobes, and the hippocampus, amygdala, caudate, putamen, globus pallidus, thalamus, anterior and posterior cingulate, substantia nigra (SN), and whole midbrain, using the automated anatomical labeling atlas implemented in PMOD software. They were modified to be devoid of CSF space using CSF maps generated from individual MRI data. Whole gray matter and whole white matter masks were also generated from individual MRI data. These VOIs were further modified to avoid high radioactivity spill-in from adjacent venous sinuses, as described previously.^b^ In addition to VOI-based quantifications of SUVRs, we performed a voxel-by-voxel jack-knife examination of parametric SUVR images using SPM12 to statistically assess distributions of areas with an increased [^11^C]PBB3 retention in each patient compared with 13 HCs.

**Supplementary References**

a. Hashimoto H, Kawamura K, Igarashi N, et al. Radiosynthesis, photoisomerization, biodistribution, and metabolite analysis of ^11^C-PBB3 as a clinically useful PET probe for imaging of tau pathology. J Nucl Med 2014;55(9):1532-1538.

b. Kimura Y, Ichise M, Ito H, et al. PET Quantification of Tau Pathology in Human Brain with ^11^C-PBB3. J Nucl Med 2015;56(9):1359-1365.

c. Ashburner J. A fast diffeomorphic image registration algorithm. Neuroimage 2007;38(1):95-113.

**
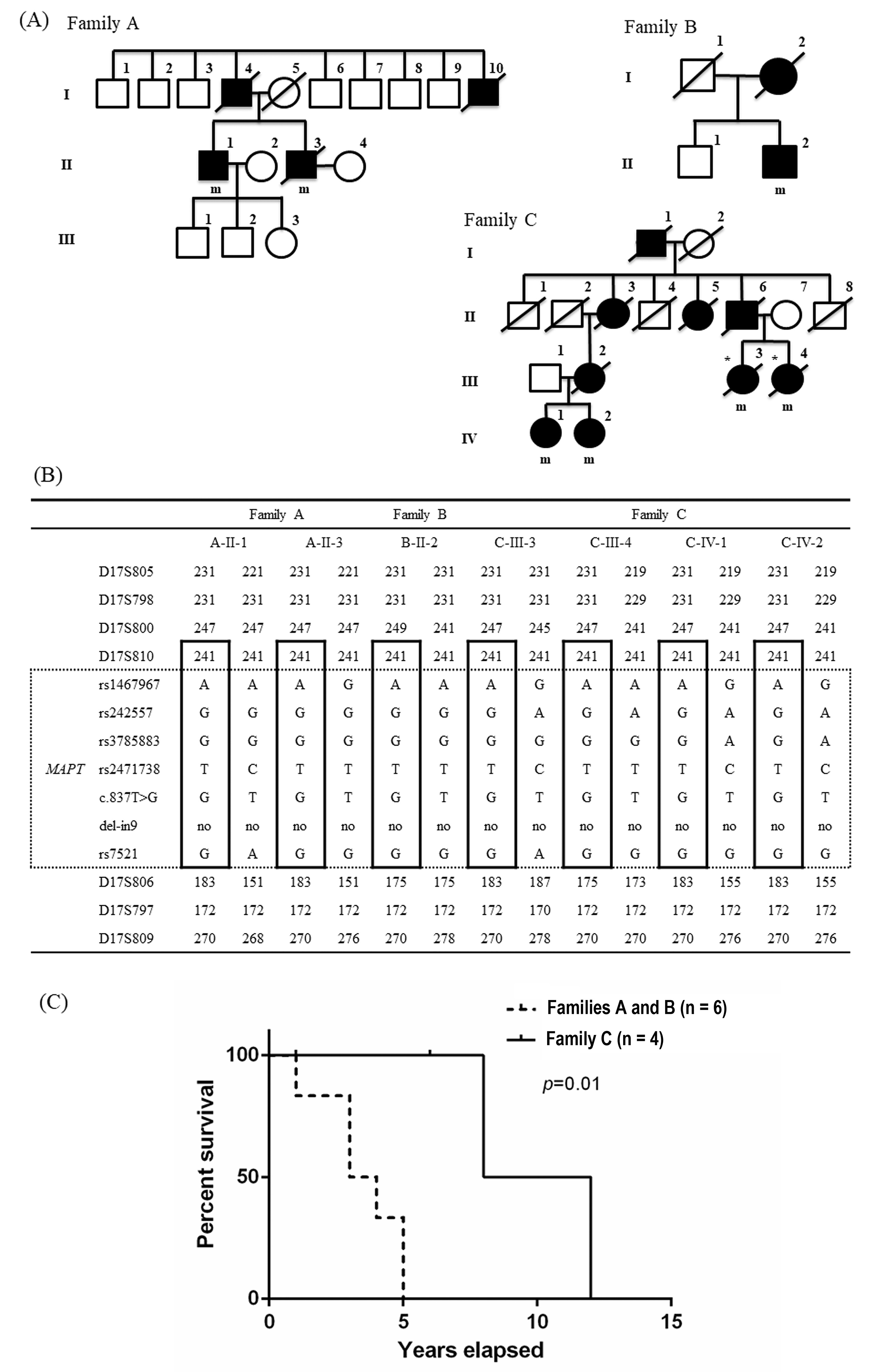
**

**Supplementary Figure 1. Genetic and clinical profiles of FTDP-17-*MAPT* patients derived from three families with the N279K *MAPT* mutation**

(A) Pedigrees of families A, B and C. Each family originated from the same rural area with autosomal dominant inheritance, manifesting young-onset Parkinsonism and progressive cognitive decline. Filled symbols denote patients with Parkinsonism and cognitive decline, while ‘m’ indicates confirmed carriers of the N279K mutation. Slashed symbols denote deceased individuals; autopsied cases are indicated by asterisks. (B) Haplotype analysis of the patients showed similar gene dosage as measured by GeneMapper and identical single nucleotide polymorphisms (SNPs) in the region of *MAPT*, indicating that all these families share a common founder. (C) Kaplan-Meier survival estimation and log-rank test for 10 patients from combined A and B families (dashed line; n = 6) and family C (solid line; n = 4) were performed using GraphPad Prism^Ⓡ^6 (GraphPad Software, Inc., San Diego, CA, USA) to compare the duration of survival after disease onset among these three families. Log-rank test indicated that families A and B exhibited shorter post-onset lifespan than family C (p = 0.01).

**Supplementary Table 1. Demography and clinical characteristics of affected members of families A, B, and C.**

|  | A-I-4 | A-I-10 | A-II-1* | A-II-3 | B-I-2 | B-II-2* | C-III-3 | C-III-4 | C-IV-1 | C-IV-2* |  |
| --- | --- | --- | --- | --- | --- | --- | --- | --- | --- | --- | --- |
| Gender | Male | Male | Male | Male | Female | Male | Female | Female | Female | Female | Male 5 (50%) |
| Age at disease onset (yrs) | 50 | 40 | 41 | 38 | 50 | 40 | 42 | 43 | 44 | 34 | 42.2 ± 4.96 |
| Age at death (yrs) | 55 | 41 | NA | 41 | 55 | 44 | 54 | 51 | NA | NA | 48.7 ± 6.50 |
| Age at examination (yrs) | NA | NA | 44 | 39 | NA | 41 | NA | NA | 44 | 40 | 41.6 ± 2.30 |
| Disease duration at examination (yrs) | 5 | NA | 3 | 1 | 5 | 1 | NA | NA | 0.5 | 6 | 3.07 ± 2.28 |
| Initial symptom at onset | Gait disturbance | Character changes | Parkinsonism | Character changes | Parkinsonism | Parkinsonism | Parkinsonism | Parkinsonism | Parkinsonism | Parkinsonism |  |
| Type of disorder | NA | NA | bvFTD | bvFTD | NA | bvFTD | PSP | PSP | NA | bvFTD |  |
| Character changes | NA | + | + | + | NA | + | + | + | + | + | 100% (8/8) |
| MMSE (/30) | NA | NA | NA | NA | NA | 23 | NA | NA | 30 | 29 |  |
| FAB (/18) | NA | NA | NA | NA | NA | 16 | NA | NA | 15 | 16 |  |
| Parkinsonism | + | + | + | + | + | + | + | + | + | + | 100% (10/10) |
| Akinesia | NA | NA | + | + | NA | + | + | + | + | + | 100% (7/7) |
| Rigidity | NA | NA | + | + | NA | + | + | + | + | + | 100% (7/7) |
| Tremor | NA | NA | + | + | NA | + | - | - | - | + | 57.1% (4/7) |
| Response for levodopa | NA | NA | - | - | NA | - | - | - | - | - | 0% (0/7) |
| Apraxia of eyelid | NA | NA | + | - | NA | - | + | + | - | - | 42.9% (3/7) |
| Abnormal eye movements | NA | NA | + | - | NA | - | + | + | - | - | 42.9% (3/7) |
| Prognosis | worsened | worsened | worsened | worsened | worsened | worsened | worsened | worsened | NA | mild |  |
| SBR of DAT scan (right/left) |  |  | 0.92/0.87 |  |  | 1.29/0.15 |  |  | 1.32/0.07 | 2.57/0.55 |  |

Cases included in a tau PET study are highlighted in light grey. Abbreviations: *, proband; MMSE, mini-mental state examination; FAB, frontal assessment battery; NA, not applicable/not available; DAT, dopamine transporter; SBR, specific binding ratio; bvFTD, behavior variant frontotemporall dementia; PSP, progressive supranuclear palsy
